# What Smoke Costs

**Published:** 1920-09-11

**Authors:** 


**THE PUBLIC WELL-BEING**—*(continued)*

### What Smoke Costs.

Almost as many estimates have been made as to the economic cost of the smoke nuisance as treatises have been written and speeches made on the discomfort and unhealthy conditions produced by smoke—though there are serious smoke advocates who seem to think that the atmosphere of a town of a thousand factory chimneys is as health-giving as the air of a mountain-top. The best known estimate of the losses arising from soot and smoke suspended in city atmosphere is probably that of £5,000,000 made twenty years ago by the Hon. Rollo Russell, who regarded this as the actual cost per annum that London had to pay for its smoke.

A correspondent of the *Manchester Guardian*,

**THE PUBLIC WELL-BEING—(*continued*).**

however, quotes as quite the most elaborate calculation of the kind one that has appeared in the form of a bulletin published by the Mellor Institute of Industrial Research in the University of Pittsburg, U.S.A. By means of funds specially given for the purpose by an anonymous donor, a staff of twenty-eight specialists was engaged to investigate the nature and extent of the damage and losses arising from smoke in and around the city of Pittsburg and the causes of and remedy for the nuisance. On this staff were eight physicians, five architects, four engineers, two chemists, two economists, a bacteriologist, a physicist, and an attorney.

The fourth of seven bulletins recently published contains the economist's estimate of the losses due to smoke in Pittsburg. The total loss is put down at £1,988,950, made up as follows:—

Cost to the smoke-maker by imperfect combustion, £304,150; cost to the individual (laundry and dry-cleaning bills), £450,000; cost to the householder (painting, cleaning, and decorating), £466,400; cost to the proprietors of wholesale and retail stores (cleaning, lighting, depreciation of stock), £735,000; cost to the owners of office buildings, hotels, and hospitals, £33,400. Calculating the loss per head for the population, it equals nearly £5 per annum for every man, woman, and child in the city.

Assuming that the losses due to smoke in some of our leading English cities and towns are only one-half as large as in Pittsburg, the correspondent gives the following estimates of costs: In London, £11,305,000; Glasgow, £1,960,000; Manchester, £1,785,000; Sheffield, £1,135,000; Leeds, £1,112,500. But the assumption seems to be a very arbitrary one, and we should like to know the data on which the Pittsburg estimates are based. One is reminded of the American lady in Arnold Bennett's play "Cupid and Commonsense," who, on her first visit to the Potteries enthusiastically declared that compared with the charm of "Bursley" Pittsburg seemed like the day after washing day!

